# By the House Governor of a Northern Hospital

**Published:** 1912-01-06

**Authors:** 


*To the Editor of* The Hospital.

### BY THE HOUSE GOVERNOR OF A NORTHERN HOSPITAL.

*To the Editor of* The Hospital.

Sir,—With reference to your letter of December 2, 1911, I am very loth indeed to make any attempt to prophesy what effect the Insurance Bill will have upon voluntary hospitals; nor does it seem to me to be necessary to do so. The English voluntary hospitals do such good work and the arguments in their favour are so strong that any Government action which is found to interfere with their efficiency must be remedied almost as soon as it is proved.

The workmen of this district, for instance, so soon as they find, if they do find, that Government action is crippling their hospital—for they look upon it now with pride as *their* hospital—will make such an outcry that Government will have to provide the necessary remedy, either by a grant in aid or by a per capita grant; and it does not necessarily follow that because Government is forced to provide this grant that Government will insist upon a controlling interference with the management of the hospital to which the grant is made. I believe the voluntary hospitals are safe because the workmen know their value and because they will have "voluntary hospital" treatment at all costs. They are perfectly alive to what kind of treatment they are likely to receive under Poor Law or any development of Poor Law. But to answer your questions in the order in which they are asked.

### Categorical Answers.

(*a*) It is probable, for a time at any rate, that the hospitals will suffer financially, but to what extent it is quite impossible to say. Some private individuals will no doubt reduce their subscriptions on the ground that they have to pay for their servants, and some employers of labour may be inclined to diminish the amount they contribute. I do not know that there is any evidence to show that legacies will fall off, for legislation for the last twenty years has all tended in the same direction, and it is still legacies that keep hospitals going. I do not know in our own case that there are any signs of a falling off. We have done this year rather better than the average.

(*b*) A definite answer to question (*b*) would depend upon a definite answer to question (*a*). If we lose so much through the action of the Bill we shall, unless the loss be made good, have to close so many beds; but I do not believe that any beds will be closed, and if we can show at the end of a year's working—and all voluntary hospitals have enough capital to keep them going for more than a year—that the Bill is crippling us, Government will be obliged to make good any deficiency, for without hospital treatment—and this the Government will find out by actual experience—the Insurance Bill is but a half measure.

### Eligibility of Patients.

(*c*) So far as the eligibility of in-patients is concerned the Insurance Bill can have little effect. It does not provide hospital treatment and it is difficult to see in its provisions anything at all likely to reduce the already overwhelming number of applicants for admission. No doubt there are a few cases admitted to voluntary hospitals, owing to the fact that so much money has been spent on medicine and outside doctors that there is sometimes not enough left to pay even a consultant's fee. Under the Insurance Bill these cases will be to a certain extent helped. They will at any rate get their medicine and their medical attendance free and will have 10s. or 7s. 6d. per week, so that when it is found, for instance, that an operation is necessary, they may still be able to scrape enough money together to pay for it to be done in private. To this (probably negligible) extent perhaps the Insurance Bill may relieve hospitals so far as the in-patients are concerned.

With regard to the out-patients, the Bill ought to relieve hospitals and the hospital out-patient staff ought to be able to devote attention to giving skilled advice in really difficult cases and not be driven, as at present, to acting as club doctors on a large scale. It is not too much to say with regard to our out-patient department that a very large proportion of the patients require food rather than skilled medical and surgical treatment, and in this I do not suppose our case is at all singular. At any rate the Bill will give hospital authorities the power of referring many present applicants for relief to the insurance doctors.

### "The Insured will Come."

(*d*) Whether the insured have or have not a right to come to the hospitals makes little difference; they will come; they must come, for there is no other place provided for them. At least the Chancellor has not explained where it is. The truth of the matter seems to be that the Chancellor believes that voluntary hospitals will be forced to rise to the occasion and pull his scheme through. No doubt the voluntary hospitals will rise to the occasion; but to do so they must have money, and, as in the past, they have always got money when they have been able to show real need, so in the future they will get money. The only difference will be that it will not all come from the public as heretofore, the Government will have to act its part and make charitable grants to hospitals wherever required. And why should not the Government make charitable grants just as corporations make charitable grants and just as big water companies, etc., etc., make charitable grants without demanding any effective interference in the management; and if a Government grant means no more than a Government inspection why should hospitals fear that?

### The Outlook.

I quite agree we shall have to fight hard against any vexatious Government interference, but in this fight each hospital will have the help of the workmen of its district who have learned the value of voluntary hospitals in which they take an intelligent interest, and in the management of which they have an effective voice. If this be so, is the outlook such a very serious one? Voluntary hospitals are able to make out for themselves an overwhelming case and Government will be quite unable to give either in any new State hospitals which it may be in the mind of the Chancellor to establish or in any of the old Poor Law hospitals which it may be in his mind to bring up to date, what is given at the present time by the voluntary hospitals. In saying this I do not in the least desire to disparage any scheme that seeks to develop the present Poor Law hospitals. There is no doubt that much might be done with them, and that much material which could be utilised is going to waste. What I do wish to say is that however Government may develop these Poor Law hospitals there will still be work for the voluntary hospitals to do, and it will be precisely the same work as they have been doing for the last 150 years.

December 6, 1911. House Governor.

